# Case Report of First Angiography-Based On-Line FFR Assessment during Coronary Catheterization

**DOI:** 10.1155/2017/6107327

**Published:** 2017-08-01

**Authors:** Ran Kornowski, Hana Vaknin-Assa

**Affiliations:** Cardiac Catheterization Laboratories, Cardiology Department, Rabin Medical Center, Belinson Hospital Affiliated to the “Sackler” Faculty of Medicine, Tel Aviv University, Petah Tikva, Israel

## Abstract

Fractional flow reserve (FFR), an index of the hemodynamic severity of coronary stenoses, is derived from hyperemic pressure measurements and requires a pressure-monitoring guide wire and hyperemic stimulus. Although it has become the standard of reference for decision-making regarding coronary revascularization, the procedure remains underutilized due to its invasive nature. FFR_angio_ is a novel technology that uses the patient's hemodynamic data and routine angiograms to generate a complete three-dimensional coronary tree, with color-coded display of the FFR values at each point along the vessels. After being proven to be as accurate as invasive FFR measurements in an off-line study, this case report presents the first on-line application of the system in the catheterization lab. Here too, a high concordance between FFR_angio_ and invasive FFR was observed. In light of the demonstrated capabilities of the FFR_angio_ system, it should emerge as an important tool for clinical decision-making regarding revascularization in patients with coronary artery disease.

## 1. Introduction

In patients with stable coronary disease, stenoses severity is assessed by pressure wire-based fractional flow reserve (FFR), a major and independent predictor of lesion-related coronary outcomes, which has become the standard of reference for decision-making regarding coronary revascularization. Yet, due to the invasive nature of the procedure FFR measurements remain underutilized [1, 2]. Therefore, the ability to derive FFR values from routinely performed coronary angiography, without the need for a pressure guide wire or drug-induced hyperemic stimulus, could have an important impact on daily clinical practice.

Several image-based FFR techniques have been introduced in the past few years. Most are based on computational fluid dynamics (CFD), a method that uses numerical analysis and algorithms to calculate fluid flows within 3D structures. Their first validation tests were rather favorable but the computational complexity of CFD requires manual interaction, as well as a considerable processing time, which is problematic for “live” application in the catheterization lab. Additionally, the proposed tools usually allow for assessment of a specific lesion* only* within a chosen segment of the coronary artery [3–9].

FFR-angiography (FFR_angio_ developed by CathWorks, Ra'anana, Israel) is a novel technology providing a three-dimensional functional mapping of the coronaries. It is based on a rapid flow analysis of a dynamically derived lumped model which can assess FFR using routine angiograms. The accuracy of the FFR_angio_ system was tested and validated in a recent publication [10].

Following the acquisition of a normal angiogram, the user enters the mean aortic pressure. The coronary tree (right or left system) is reconstructed in 3D based on two or more conventional single-plane angiographic projections of the vessels, whereby epipolar ray tracing together with mathematical constraints enforcing the tree's structure is utilized. Next, the system scans the entire reconstructed tree in 3D and analyzes each branch as well as each bifurcation, looking for narrowed regions. Hyperemic flow ratio is derived from automatic resistance-based lumped mapping along the entire coronary tree. The FFR values at each point along the vessel are color-coded and superimposed on the 3D epicardial model and cut-off values of 0.80 identical to standard invasive FFR apply. FFR_angio_ does not utilize pharmacologic drug-induced hyperemia. This report describes the first “live” utilization of FFR_angio_ during an actual catheterization procedure.

## 2. Case Presentation

### 2.1. Clinical History

A 74-year-old man was referred to our hospital for diagnostic angiography. His clinical history included unstable angina, insulin-dependent diabetes mellitus, past smoking, dyslipidemia, and mild renal insufficiency. The patient underwent coronary catheterization the previous year due to stable angina; multiple moderate lesions were detected in the Left Anterior Descending (LAD) and a conservative treatment strategy was then recommended.

### 2.2. Case Description and Diagnosis

**Figure 1:**
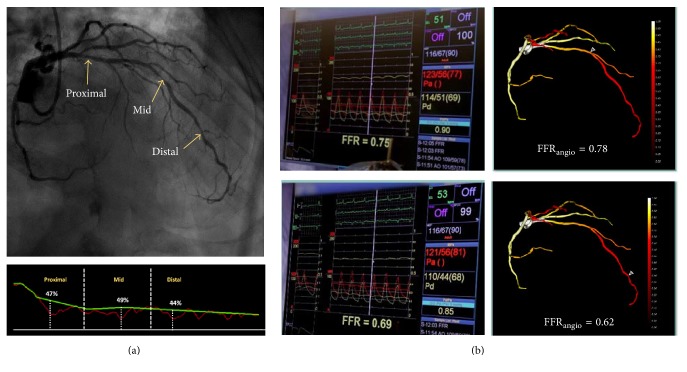
(a)* (Top)* LAD lesions (arrows indicate the proximal, mid, and distal lesions) and* (bottom)* corresponding percent diameter stenosis used for diagnostic FFR_angio_ quantitative coronary angiography (QCA) calculation. (b) Invasive fractional flow reserve (FFR) measurements* (left)* of the middle (top) and distal (bottom) lesions and the corresponding values calculated by the FFR_angio_ system (right).

After 1 year, the patient was returned to the catheterization lab with NSTEMI. Diagnostic angiography revealed three consecutive discrete stenoses located in the proximal, middle, and distal segments of the LAD without evidence of diffuse disease (Figure 1(a)), and the patient was included as part of a 53-patient single-center study examining the accuracy of FFR_angio_ when used on-line during the procedure. Based on visual estimation and the operator's decision, invasive FFR was performed for the middle and distal lesions only. Invasive fractional flow reserve (FFR) measurements were 0.75 and 0.69, respectively (Figure 1(b)).

### 2.3. FFR_angio_

The FFR_angio_ algorithm was applied in parallel to the invasive measurements. A 3D quantitative coronary angiography analysis (QCA) was derived and yielded diameter stenoses of 49% and 44% for the middle and distal LAD lesions, respectively. The system created a 3D image of the coronary tree showing the calculated FFR_angio_ values along the branches according to a color-coded scale. The calculated FFR_angio_ values at the exact locations of the invasive measurements (e.g., using a pressure wire) were 0.78 and 0.62 for the middle and distal lesions, respectively. Figure 1(b) presents the 3D QCA analysis as well as the invasive FFR and corresponding computed values obtained by the FFR_angio_ measurements for the middle and distal stenoses.

### 2.4. Poststenting FFR Evaluation

**Figure 2:**
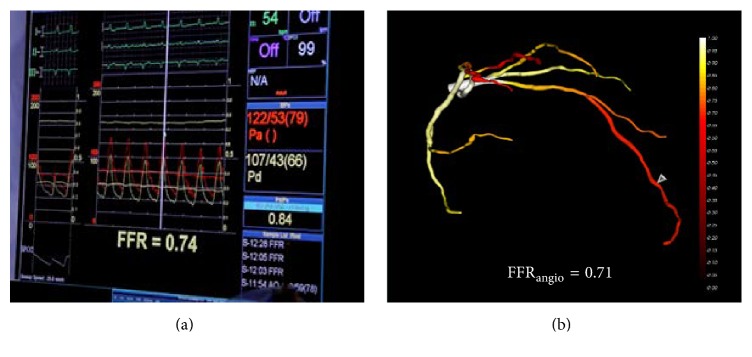
Posttreatment FFR (a) and FFR_angio_ (b) assessments at the distal LAD segment following stenting of the middle and distal lesions.

Following stenting of the middle and distal LAD lesions, invasive FFR measurement at the distal LAD segment was 0.74. Image-based FFR_angio_ values at the distal LAD were calculated once again by using the posttreatment angiography, yielding a value of 0.71, and compared to the invasive FFR value (Figure 2). As both FFR values remained significant (<0.8) after stenting the middle and distal stenoses, the proximal LAD lesion was stented as well.

## 3. Discussion

The FFR_angio_ system that was utilized in our case is a novel image-based technology that enables an image-based assessment of FFR, calculated from common angiograms reconstructed in 3D along with routinely measured hemodynamic data. Importantly, the FFR_angio_ system, which is not based on CFD, was designed to keep the pace of the catheterization procedure; that is, the time from image acquisition to display of the FFR_angio_ results is <10 min in most cases and the entire procedure requires minimal input from the operator.

In the case presented herein, the FFR_angio_ system detected the two lesions seen in the 2D angiography which were evaluated with invasive FFR and successfully performed a lesion-per-lesion evaluation of the coronary physiology, without necessitating fragmentation of the measurements. Thus, the impact of the stenoses on the coronary tree was fully presented to the operator as a physiological roadmap. With the fact that the FFR_angio_ system is considered an investigational device, only the invasive FFR was used to guide treatment.

While other groups have attempted to use angiographic data to simulate invasive FFR measurements, [3–6, 8, 9, 11, 12], the methods are laborious, require additional analysis, and often involve long processing times which are limited to off-line analysis by a core laboratory. Of special interest is the quantitative flow ratio (QFR) method. Tu et al. used the QFR method derived from 3 different flow models (QAngio XA 3D prototype and Medis, Leiden, Netherlands) based on 3D QCA of vessel segments and the flow moving through the stenosis [8]. This system relies on 3D QCA combined with additional TIMI frame count from high-quality images (30 frames/second) for the calculation of mean volumetric flow rate at hyperemia and still requires induced hyperemic conditions. This method also provides assessment of the main vessel of interest without providing the side branches.

## 4. Conclusion

In summary, we have demonstrated for the first time a coronary case utilizing “on-line” FFR_angio_ technology, showing good concordance with the wire-based FFR in sequential lesions of the LAD both before and after stenting. The system allowed for comprehensive functional evaluation of the vessel without the need for drug-induced hyperemia and/or fragmentation of the measurements, as required using conventional invasive FFR. Once confirmed in larger studies and on a wide spectrum of coronary lesions, FFR_angio_ should emerge as an important tool for physiologic assessment of coronary artery disease.
